# Do Effort and Reward at Work Predict Changes in Cognitive Function? First Longitudinal Results from the Representative German Socio-Economic Panel

**DOI:** 10.3390/ijerph14111390

**Published:** 2017-11-15

**Authors:** Natalie Riedel, Johannes Siegrist, Natalia Wege, Adrian Loerbroks, Peter Angerer, Jian Li

**Affiliations:** 1 Institute of Occupational, Social, and Environmental Medicine, Centre for Health and Society, Faculty of Medicine, University of Düsseldorf, 40225 Düsseldorf, Germany; natalia.wege@uni-duesseldorf.de (N.W.); adrian.loerbroks@uni-duesseldorf.de (A.L.); peter.angerer@uni-duesseldorf.de (P.A.); 2 Department of Social Epidemiology, Institute of Public Health and Nursing Research, University of Bremen, 28359 Bremen, Germany; 3 Senior Professorship on Work Stress Research, Life Science Center, Faculty of Medicine, University of Düsseldorf, 40225 Düsseldorf, Germany; johannes.siegrist@med.uni-duesseldorf.de; 4 Department of Psychiatry and Psychotherapy, Faculty of Medicine, University of Düsseldorf, 40225 Düsseldorf, Germany

**Keywords:** effort–reward imbalance model, cognitive function, working population, longitudinal analysis, Socio-Economic Panel

## Abstract

It has been suggested that work characteristics, such as mental demands, job control, and occupational complexity, are prospectively related to cognitive function. However, current evidence on links between psychosocial working conditions and cognitive change over time is inconsistent. In this study, we applied the effort–reward imbalance model that allows to build on previous research on mental demands and to introduce reward-based learning as a principle with beneficial effect on cognitive function. We aimed to investigate whether high effort, high reward, and low over-commitment in 2006 were associated with positive changes in cognitive function in terms of perceptual speed and word fluency (2006–2012), and whether the co-manifestation of high effort and high reward would yield the strongest association. To this end, we used data on 1031 employees who participated in a large and representative study. Multivariate linear regression analyses supported our main hypotheses (separate and combined effects of effort and reward), particularly on changes in perceptual speed, whereas the effects of over-commitment did not reach the level of statistical significance. Our findings extend available knowledge by examining the course of cognitive function over time. If corroborated by further evidence, organization-based measures in the workplace can enrich efforts towards preventing cognitive decline in ageing workforces.

## 1. Introduction

With increasing life expectancies, dementia has advanced as a major concern to public health worldwide [1,2,3]. In Western Europe, the prevalence of dementia has been recently estimated at nearly 7% among the population aged over 60 years and described as exponential function of age from this baseline age onwards [1,2,4]. As there is still no aetiology-specific treatment available [3,4], knowledge on amenable risk factors is of utmost importance for effective primary prevention [5]. Besides cardiovascular risk factors related to behavior (e.g., smoking, physical inactivity, and obesity in mid-life) and associated chronic conditions (e.g., hypertension and diabetes), education, and cognitive activities have been identified as promising entry points for primary prevention of dementia across adulthood [3,5,6,7].

Neuro-cognitive approaches such as “differential preservation” [8], “cognitive reserve” [9,10], or “complexity of work environment” [11] have been proposed to exert protective effects on cognitive function among older populations [12]. Biologically, it is plausible to assume that stimulating cognitive activities enhance the plasticity of neuronal circuits enabling the brain to delay age-related cognitive decline and pathological changes [13,14]. This assumption is referred to as the “use it or lose it” hypothesis. One major field associated with cognitive activity in adulthood relates to one’s occupational tasks. Therefore, ageing research in the field of occupational health psychology and epidemiology has primarily focused on the effects of distinct mental task features, such as “complexity,” and “novelty” on cognitive function [15,16]. These psychosocial work characteristics were also framed and operationalized as job demands (time pressure and required concentration) as well as job control (skill discretion, task variety, learning opportunities, decision latitude, and self-directedness) in line with Karasek’s well-known “demand–control” model [17]. Accordingly, higher levels of job demands and job control are assumed to be conducive to employees’ cognitive health. Joint exposure to both high job demands and control constitutes an “active job,” according to the model, fostering learning and strengthening brain capacities through neuro-cognitive stimulation. Findings from most prospective studies on mental work demands in general [11,18,19,20] as well as on job demands and job control in particular [21,22,23,24,25] tentatively or partly support the “use it or lose it” hypothesis. However, in a recent systematic review, the overall evidence on the link of working characteristics and cognitive decline has been rated as “insufficient, conflicting or weak” [15].

In this study, we complemented the work-related neuro-cognitive approaches by a neuro-affective perspective focusing on reward processing and reward-based learning. Distinct processes within the brain reward circuits are involved in cognitive performance [26,27]. For instance, positive learning reward expectancies are linked to dopaminergic neurons [28], the deficiency of which may account for impaired information processing and reduced working memory. Behavioral and motor abnormalities related to these deficiencies were observed in patients with Alzheimer or frontotemporal dementia [26,29]. Conversely, an inclination to positive affectivity in reward-based learning has been conceived as a feature of healthy ageing [26]. In the occupational context, receiving reward strongly builds on social interactions with colleagues and superiors and is usually related to successful accomplishment of tasks, thus eliciting positive emotions of self-esteem and enhancing work-related motivation, with beneficial effects on cognitive attention, stimulation, and performance [30]. Along these lines, we hypothesize that a psychosocial work environment that supports learning processes by offering positive rewards such as promotion prospects, recognition, and esteem may preserve cognitive function of working people from age-related decline, or at least decelerate its initiation. To our knowledge, this hypothesis has not yet been tested in the context of occupational health research, although it may be implicated in the concept of complex work environments [11] and in research on work motivation, learning, and development in an ageing workforce [12].

In order to accomplish a reward-based extension of previous research, we applied Siegrist’s effort–reward imbalance model to this analysis [31]. As a complementary concept to Karasek’s demand–control model mentioned above, this model focuses on the work contract and its core principle of reciprocity between efforts spent and rewards received in return. In addition, it includes an intrinsic component in terms of a coping pattern termed “over-commitment” (inability to withdraw from work at the expense of recovery) [32]. In the present contribution, we aimed to test longitudinal associations of psychosocial work environment measured by this model with cognitive function, using a representative sample of the German working population. Specifically, we expected that high effort, high reward, and an absence of excessive over-commitment were positively associated with cognitive changes. Importantly, a favorable balance of effort and reciprocated rewards is assumed to contribute to cognitive improvement in addition to the single components mentioned.

## 2. Materials and Methods

### 2.1. Study Population and Study Sample

Our study is based on the German Socio-Economic Panel (GSOEP) that is coordinated by the German Institute for Economic Promotion (Deutsches Institut für Wirtschaftsforschung, DIW), Berlin. This well-established longitudinal study covers a randomly selected and representative sample of the German adult population aged 18+. Since its initiation in 1984, this study has been followed up and replenished annually [33,34]. Participants gave their informed consent prior to data collection. Compliant with national laws as well as evaluated and approved by the German Council of Science and Humanities (Wissenschaftsrat), the GSOEP is ethically sound and explicitly intended for epidemiological analyses [34].

We used data from two waves in 2006 and in 2012. In 2006, the GSOEP included a total of 22,358 participants. Complete information on psychosocial work characteristics including the effort–reward imbalance model and other relevant covariates (see below) was available for 9746 employed participants. Given that cognitive function is assumed to be modifiable throughout adulthood, we did not restrict the age range within the employed population.

**Figure 1:**
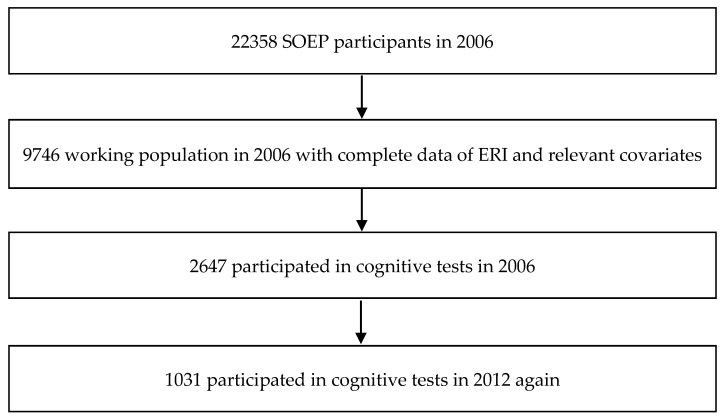
Flow chart of the study sample.

Cognitive tests (i.e., perceptual speed and verbal fluency, see below for details) were conducted in random sub-samples in both waves of 2006 and 2012, yielding a sample of 1031 employed participants with repeated measurements of perceptual speed and verbal fluency (see Figure 1).

### 2.2. Exposure and Outcome Measures, Covariates

The validated short version of the Effort–Reward Imbalance questionnaire was applied in the GSOEP 2006, with three items for “effort” (e.g., “Over the past few years, my job has become more and more demanding”), seven items for “reward” (e.g., “I receive the respect I deserve from superior or a respective relevant person,” “Considering all my efforts and achievements, my job promotion prospects are adequate,” and “My job security is poor”), and six items for “over-commitment” (e.g., “People close to me say I sacrifice too much for my job” and “Work rarely lets me go, it is still on my mind when I go to bed”) (please see [35] for details). Responses to the items of “effort” and “reward” were scored on a 5-point scale where a value of 1 indicated no respective distressful experience, and a value of 5 indicated very high distressful experiences. The items of “over-commitment” were scored on a 4-point scale (1 = full disagreement, 4 = full agreement with statement). Sum scores were computed with higher scores reflecting higher effort, reward, and over-commitment with potential scoring ranges of 3–15, 7–35, and 6–24, respectively. The psychometric properties of this questionnaire were tested in the GSOEP sample, with satisfactory reliability (Cronbach’s alpha coefficients were 0.74, 0.79, and 0.79 for “effort,” “reward,” and “over-commitment,” respectively), as well as good factorial, discriminant, and criterion validity. Median cut-off points were used to compare high levels with low levels of effort, reward, and over-commitment. In line with the theoretical model, the “reward” scale was further divided in its three sub-scales “esteem,” “promotion prospects,” and “job security” [31,35]. In addition, we built a composite variable from the binary variables for effort and reward, with low levels of both effort and reward as reference category.

It is generally not feasible to perform comprehensive neuropsychiatric assessments in large-scale epidemiological studies. Therefore, two short well-established and validated tests of cognitive ability with slight modification were used in the GSOEP by means of Computer Assisted Personal Interviewing (CAPI). These were the Symbol-Digit Test (SDT) and Animal Naming Test (ANT) [36]. These two tests have been widely used in several large epidemiological studies, e.g., [18,21,25,37]. The SDT measures cognitive mechanics that are hard-wired, biologically based capacities for information processing. This test corresponds to a sub-module in the non-verbal section of the Wechsler Adult Intelligence Scale (WAIS) and examines cognitive processing speed, accuracy, processing capacity, coordination, and inhibition of basic cognitive processes, representing perceptual speed, working memory, and the capacity for deductive reasoning. The ANT measures cognitive pragmatics, i.e., training-related competencies. This test corresponds to a sub-module in the verbal section of the WAIS and represents verbal fluency. Recently, the cognitive tests in GSOEP have been successfully applied to study healthy aging [38]. To measure the changes in perceptual speed and word fluency between 2006 and 2012, test scores in 2006 were subtracted from test scores in 2012. Thus, positive score values indicated an improved cognitive function.

In addition, demographic (age, gender, and marital status), socioeconomic (education), lifestyle behaviors (smoking, alcohol consumption, and body mass index), and a short summary measure of health functioning (SF-12 physical and mental health) were also collected in 2006. These variables were included in statistical multivariate analyses as covariates. For instance, behavioral risk factors have been linked to work stress and dementia [3,5,39].

### 2.3. Statistical Analyses

Multivariate linear regressions were used to assess the associations of effort, reward, and their combinations, as well as over-commitment, with the changes in perceptual speed and verbal fluency between 2006 and 2012, respectively. Sociodemographic variables (age, gender, marital status, and education) and the score of respective cognitive tests at our baseline year in 2006 were regarded as a basic adjustment set (Model I). Robustness of associations was examined in two subsequent models that included behavioral risk factors of dementia (smoking, alcohol drinking, and BMI in 2006) in the first step (Model II) and physical and mental health in the second step (Model III).

## 3. Results

Characteristics of our study sample are given in Table 1. Both genders were equally distributed and two-thirds were married. A relatively high mean level of formal education (ca. 13 years) may reflect a characteristic of the German system that combines mandatory school education with vocational training. Overweight was the most common behavioral risk factor (52%). Smoking and regular alcohol drinking were reported by 32% and 17% of the participants, respectively. Distributions of physical and mental health scales were fairly similar, with a mean value of about 53 each. Median cut-off points of effort, reward, and over-commitment (7, 31, and 13, respectively) roughly corresponded to the respective mean values shown in Table 1. On average, in this age group under study (mean 44 years old), performance on the two cognitive tests (perceptual speed and verbal fluency) slightly improved from 2006 to 2012.

Results from multivariate linear regressions on changes in perceptual speed and verbal fluency are displayed in Table 2 and Table 3, respectively.

In Table 2, high levels of effort and reward at work in 2006 were each significantly associated with an increase in perceptual speed between 2006 and 2012, with estimated mean effects of effort (b = 1.90) being slightly higher than those of reward (b = 1.73) in Model I. According to the theoretical model, three components of reward have additionally been included in this analysis, and the component “promotion prospects” exerted a significant effect. In addition, in comparison to the reference group characterized by low effort and low reward levels, the remaining combinations of these two extrinsic components of the model were associated with significant improvements in perceptual speed over 6 years, with the relatively most pronounced effect for the combination “high effort and high reward” (b = 3.24). Patterns remained largely unaffected by adjustment for additional covariates in Models II and III. Concerning the model’s intrinsic component over-commitment, we did not observe a significant effect, despite an elevated coefficient in Model III.

In Table 3, the respective results are given for changes in verbal fluency between 2006 and 2012. Different from perceptual speed, high reward, but not high effort, emerged as a significantly positive predictor of improved cognitive functioning in this analysis (b = 2.09, in Model III). Again, the reward subscale of “promotion prospects” yielded a significant effect. Concerning the combination of the two extrinsic components of the model, the co-manifestation of high effort and high reward was the only variable category with a significant effect (in the fully adjusted Model III). The intrinsic component over-commitment was not significantly associated with improved verbal fluency.

## 4. Discussion

In this study, we tested the hypothesis that components of the model of effort–reward imbalance at work are positively related to improvements of cognitive function over a 6-year observation period in a representative middle-aged working population in Germany. More specifically, high effort and high reward, as well as a lack of excessive over-commitment to work, were expected to yield significant effects on the two measures of cognitive function under study, perceptual speed and verbal fluency. While high effort and high reward, both as separate and combined variables, were positively associated with perceptual speed, high reward and the co-manifestation of high effort and high reward only were related to verbal fluency. In both analyses, the subcomponent “promotion prospect” was the important aspect within the reward construct. Concerning the model’s intrinsic component over-commitment, our hypothesis was not confirmed. In additional sensitivity analyses, using the upper tertile of scale scores as an alternative cut-off point to define high vs. low levels of psychosocial working conditions yielded a very similar pattern of associations between effort–reward imbalance and changes in cognitive function (data are available upon request). Restricting the analyses to participants working at both time points in 2006 and 2012 did not produce different patterns as well (data are available upon request). The results of this study lend some preliminary support to the notion that positive experience of reward in response to effort spent at work may be conducive to cognitive function, given the links between affective and cognitive information processing in the brain reward circuits.

When relating our findings to the current literature, it is easier to discuss the role of effort, given that reward at work, our innovative feature, has not yet been studied previously in this context. The operationalization of “effort” is closely linked to the notion of high mental work demand, a notion that is also underlying the measurement of “demand” in Karasek’s job demand–control model. In our study, a significant association of effort with cognitive functioning was restricted to perceptual speed, while it was unrelated to verbal fluency. However, we need to be cautious in interpreting a single statistically significant effect, in particular in view of the fact that in two longitudinal investigations on associations of job demands with perceptual speed the relationship turned out to be inverse [25,40], at least after adjustment for education and occupation [25]. A further study finding deserves attention. When looking at all the results of Table 2 and Table 3, we observed that associations of psychosocial work characteristics with cognitive improvement were less consistent in cases of the verbal fluency test (Animal Naming Test) compared to the perceptual speed test (Symbol-Digit Test). It is possible to explain this inconsistency by the fact that different cognitive domains or abilities are measured by the two tests. Perceptual speed primarily reflects fluid cognitive abilities, while verbal fluency, as measured by the Animal Naming Test, is conceived as an interplay of fluid and crystallized cognitive abilities [41,42]. Whether these two types of cognitive abilities are related in different ways to beneficial work-related conditions, such as high effort and high reward, deserves more detailed analysis in future research. 

Taken together, the associations of reward at work with the two measures of cognitive function were somewhat more consistent than those with effort, and it was evident that reward in terms of promotion prospects contributed to this consistency. This finding lends some preliminary support to the assumption that neuro-affective processes related to the reward system may provide an additional link between recurrent beneficial experiences at work and cognitive functioning. In view of the mean age of the study population (44 ± 10 years at baseline), occupational promotion prospects may matter more than esteem reward, and more than job security, given a low level of unemployment in Germany during the observation period. We have no explanation why no support was found for the over-commitment hypothesis. According to the theoretical argument, excessive striving at work in combination with a high motivation of being in control of one’s proximal environment entails the risk of cognitive misperception (e.g., under-estimation of demands) and cognitive rigidity [31]. Thus, scoring low on over-commitment is expected to contribute to more accurate perceptual and computational functioning. However, given the score distribution with a low mean level of over-commitment in this sample (mean 13.0 ± 3.9), an explanatory role of this variable may be compromised.

The fact that mean scores of cognitive function over time slightly improved rather than declined in this middle-aged cohort deserves an additional comment. One explanation points to a retest bias in repetitive cognitive testing. Yet, as we adjusted for baseline cognitive function and as the time interval between the two tests was 6 years, this explanation is not convincing. The “Flynn effect” may offer an alternative explanation as it captures the observation of an “age gain” of enhanced cognitive function across generations and even between cohorts with relatively small age differences. Along this hypothesis, some evidence was observed in a recent publication based on GSOEP data, comparing cognitive function (Symbol Digit Test) between two different age groups within a sample of elderly people (50–90 years). Here, an “age gain” of five to eight years in test performance has been demonstrated [43].

This study suffers from a number of limitations. First, the analysis is restricted to two waves of data collection. A robust test of changes over time in cognitive function would require additional measurement waves. This limitation also applies to the measurement of work stress. As we have no information on stressful work in 2012, no statement about a potential bi-directionality of effects can be made. Second, the GSOEP study included just two domains of cognitive function that may reflect different degrees of fluid intelligence or facets of crystalline intelligence, perceptual speed and verbal fluency. Other relevant aspects, such as phonemic fluency, episodic memory, inductive reasoning, or executive function were not included [21,23,24,25,40]. Third, we focused this study on one out of several available theoretical concepts of a stressful psychosocial work environment [17,21,25,44]. While the choice of the effort–reward imbalance model [31] has been justified by an innovative research hypothesis, and while respective data were available in 2006, we may nevertheless have bypassed other qualitative aspects of a health-conducive work environment with relevance to cognitive function. Fourth, as no clinical data on mental and physical disorders or their main risk factors were available, their potential confounding impact on cognitive function could not be assessed. Obviously, a self-reported measure of current health status, as available in the GSOEP data of 2006, cannot rule out this limitation. Fifth, we could not preclude selection occurring between 2006 and 2012: Participants who did not participate in cognitive tests in 2012 again were significantly younger than those who underwent the testing a second time and were included in our study (see Figure 1). Given the strong association of cognitive impairment with older age, it is unlikely that this selection has biased the general research results. Finally, the clinical significance of our results must be judged as modest. We observed a slight overall increase in the two tests of cognitive function within a middle-aged working population over a time span of six years. Whether we can expect a potential impact of favorable psychosocial working conditions on the postponement of cognitive decline in older ages remains largely unknown.

There are also several strengths in this study. Different from several previous reports dealing with associations of demand and control at work and cognition [15], in our analysis we were able to take the baseline values of cognitive function into account. Moreover, our study population has been recruited from a nationally representative sample of working people, whereas several earlier reports were based on employees from specific companies or organizations (e.g., [21,25]). Given the documented influence of several sociodemographic and socioeconomic factors on cognitive function, our study adjusted for these factors in stepwise multivariate model testing. Furthermore, this study applied a validated standard measurement of an internationally established theoretical concept of stressful work, effort–reward imbalance, whose success in explaining work-related health outcomes has been widely documented [45].

## 5. Conclusions

To conclude, our study is one of the first to document a positive association of balanced exchange between high effort and high reward at work with improved cognitive function over a 6-year period among a nationally representative group of middle-aged men and women in Germany, where cognitive function was assessed by perceptual speed and verbal fluency. If corroborated by further evidence, our results can instruct organization-based measures of maintaining and improving a health-conducive work environment that may contribute to the prevention of cognitive decline in ageing workforces.

## Figures and Tables

**Table 1:** Characteristics of study subjects (*n* = 1031).

Characteristics		
**Continuous Variables**		**Mean ± SD**
Age in 2006	Years	44.44 ± 10.08
Education in 2006	Years	12.66 ± 2.72
Physical health (SF-12) in 2006		52.92 ± 8.11
Mental health (SF-12) in 2006		53.29 ± 7.90
Effort in 2006		7.06 ± 3.13
Reward in in 2006		29.55 ± 4.99
Esteem reward in 2006		8.77 ± 1.87
Promotion reward in 2006		11.99 ± 2.57
Security reward in 2006		8.78 ± 2.08
Over-commitment in 2006		13.04 ± 3.94
Perceptual speed in 2006		25.20 ± 13.35
Perceptual speed in 2012		31.44 ± 8.29
Word fluency in 2006		27.87 ± 10.70
Word fluency in 2012		30.37 ± 11.10
**Categorical Variables**		**N (%)**
Gender in 2006	Men	513 (49.76%)
	Women	518 (50.24%)
Marital status in 2006	Married	697 (67.61%)
	Single	182 (17.65%)
	Separated, divorced, widowed	152 (14.74%)
Smoking in 2006	No	697 (67.60%)
	Yes	334 (32.40%)
Alcohol drinking in 2006	Occasionally, seldom, never	854 (82.83%)
	Regularly	177 (17.17%)
BMI in 2006	Normal (<25)	495 (48.01%)
	Overweight (≥25 and <30)	362 (35.11%)
	Obese (≥30)	174 (16.88%)

**Table 2:** Associations of ERI in 2006 with changes in Perceptual Speed (Symbol-Digit Test) during 2006–2012 (*n* = 1031).

		Model I	Model II	Model III
Effort	Low	0	0	0
	High	1.90 (0.51, 3.30) **	1.94 (0.54, 3.33) **	1.88 (0.47, 3.30) **
Reward	Low	0	0	0
	High	1.73 (0.45, 3.01) **	1.57 (0.28, 2.86) *	1.64 (0.34, 2.94) *
Esteem reward	Low	0	0	0
	High	0.89 (−0.40, 2.18)	0.78 (−0.51, 2.08)	0.82 (−0.48, 2.13)
Promotion reward	Low	0	0	0
	High	1.68 (0.41, 2.94) **	1.55 (0.28, 2.82) *	1.62 (0.33, 2.91) *
Security reward	Low	0	0	0
	High	1.30 (−0.03, 2.64)	1.20 (−0.13, 2.54)	1.22 (−0.13, 2.57)
Over-commitment	High	0	0	0
	Low	0.75 (−0.60, 2.10)	0.78 (−0.57, 2.12)	0.87 (−0.51, 2.24)
E-R Combinations	Low Effort + Low Reward	0	0	0
	High Effort + Low Reward	2.69 (0.94, 4.43) **	2.77 (1.03, 4.52) **	2.72 (0.95, 4.49) **
	Low Effort + High Reward	2.50 (0.86, 4.15) **	2.39 (0.74, 4.04) **	2.44 (0.78, 4.10) **
	High Effort + High Reward	3.24 (1.08, 5.41) **	3.10 (0.93, 5.26) **	3.11 (0.94, 5.28) **

Multivariate linear regression, * *p* < 0.05, ** *p* < 0.01. Model I: adjusted for age, gender, marital status, education, and perceptual speed in 2006. Model II: Model I + additionally adjusted for smoking, alcohol drinking, and BMI in 2006. Model III: Model II + additionally adjusted for physical health and mental health in 2006.

**Table 3:** Associations of ERI in 2006 with changes in verbal fluency (Animal Naming Test) during 2006–2012 (*n* = 1031).

		Model I	Model II	Model III
Effort	Low	0	0	0
	High	1.60 (−0.53, 3.73)	1.61 (−0.52, 3.74)	1.69 (−0.22, 3.61)
Reward	Low	0	0	0
	High	1.82 (−0.13, 3.76)	1.83 (−0.13, 3.79)	2.09 (0.34, 3.84) *
Esteem reward	Low	0	0	0
	High	0.49 (−1.47, 2.45)	0.55 (−1.42, 2.52)	0.85 (−1.11, 2.81)
Promotion reward	Low	0	0	0
	High	1.73 (−0.18, 3.63)	1.79 (−0.13, 3.72)	2.01 (0.08, 3.94) *
Security reward	Low	0	0	0
	High	0.42 (−1.61, 2.44)	0.41 (−1.62, 2.44)	0.72 (−1.31, 2.76)
Over-commitment	High	0	0	0
	Low	0.64 (−1.42, 2.71)	0.73 (−1.34, 2.81)	1.65 (−0.24, 3.54)
E-R Combinations	Low Effort + Low Reward	0	0	0
	High Effort + Low Reward	0.65 (−2.02, 3.32)	0.62 (−2.07, 3.30)	1.42 (−1.00, 3.85)
	Low Effort + High Reward	0.86 (−1.67, 3.39)	0.83 (−1.73, 3.39)	1.82 (−0.46, 4.11)
	High Effort + High Reward	3.82 (0.50, 7.14) *	3.84 (0.51, 7.17) *	3.88 (0.93, 6.84) *

Multivariate linear regression, * *p* < 0.05. Model I: adjusted for age, gender, marital status, education, and perceptual speed in 2006. Model II: Model I + additionally adjusted for smoking, alcohol drinking, and BMI in 2006. Model III: Model II + additionally adjusted for physical health and mental health in 2006.
